# Managing heterogeneity in the study of neural oscillator dynamics

**DOI:** 10.1186/2190-8567-2-5

**Published:** 2012-03-14

**Authors:** Carlo R Laing, Yu Zou, Ben Smith, Ioannis G Kevrekidis

**Affiliations:** 1 Institute of Information and Mathematical Sciences, Massey University, Private Bag 102-904, North Shore Mail Centre, Auckland, 0745, New Zealand; 2 Department of Chemical and Biological Engineering and Program in Applied and Computational Mathematics, Princeton University, Princeton, NJ, 08544, USA; 3 Research Centre for Cognitive Neuroscience, Department of Psychology, University of Auckland, Private Bag 92019, Auckland, New Zealand

**Keywords:** heterogeneity, neural oscillators, pre-Bötzinger complex, model reduction, bifurcation, computation

## Abstract

We consider a coupled, heterogeneous population of relaxation oscillators used to model rhythmic oscillations in the pre-Bötzinger complex. By choosing specific values of the parameter used to describe the heterogeneity, sampled from the probability distribution of the values of that parameter, we show how the effects of heterogeneity can be studied in a computationally efficient manner. When more than one parameter is heterogeneous, full or sparse tensor product grids are used to select appropriate parameter values. The method allows us to effectively reduce the dimensionality of the model, and it provides a means for systematically investigating the effects of heterogeneity in coupled systems, linking ideas from uncertainty quantification to those for the study of network dynamics.

## 1 Introduction

 Networks of coupled oscillators have been studied for a number of years [1-7]. One motivation for these studies is that many neurons, when isolated (and possibly injected with a constant current), either periodically fire action potentials [8,9] or periodically move between quiescence and repetitive firing (the alternation being referred to as bursting [10,11]). In either case, the isolated neuron can be thought of as an oscillator. Neurons are typically coupled with many others via either gap junctions [12] or chemical synapses [13-15]; hence, a group of neurons can be thought of as a network of coupled oscillators.

 As an idealisation, one might consider identical oscillators; in which case, the symmetry of the network will often determine its possible dynamics [16,17]. However, natural systems are never ideal, and thus, it is more realistic to consider *heterogeneous* networks. Also, there is evidence in a number of contexts that heterogeneity within a population of neurons can be beneficial. Examples include calcium wave propagation [18], the synchronisation of coupled excitable units to an external drive [19,20], and the example we study here: respiratory rhythm generation [13,21].

 One simple way to incorporate heterogeneity in a network of coupled oscillators is to select one parameter which affects the individual dynamics of each oscillator and assign a different value to this parameter for each oscillator [3,15,22,23]. Doing this raises natural questions such as from which distribution should these parameter values be chosen, and what effect does this heterogeneity have on the dynamics of the network?

Furthermore, if we want to answer these questions in the most computationally efficient way, we need a procedure for selecting a (somehow) optimal representative set of parameter values from this distribution. In this paper, we will address some of these issues.

In particular, we will show how - given the distribution(s) of the parameter(s) describing the heterogeneity - the representative set of parameter values can be chosen so as to accurately incorporate the effects of the heterogeneity without having to fully simulate the entire large network of oscillators.

 We investigate one particular network of coupled relaxation oscillators, derived from a model of the pre-Bötzinger complex [13,14,24], and show how the heterogeneity in one parameter affects its dynamics. We also show how heterogeneity in more than one parameter can be incorporated using either full or sparse tensor product grids in parameter space.

 Our approach thus creates a bridge between computational techniques developed in the field of uncertainty quantification [25,26] involving collocation and sparse grids on the one hand, and network dynamics on the other. It also helps us build accurate, reduced computational models of large coupled neuron populations.

 One restriction of our method is that it applies only to states where all oscillators are synchronised (in the sense of having the same period) or at a fixed point. Synchronisation of this form typically occurs when the strength of coupling between oscillators is strong enough to overcome the tendency of non-identical oscillators to desynchronise due to their disparate frequencies [2,3,27] and is often the behaviour of interest [6,13,14,23].

We present the model in Section 2 and show how to efficiently include parameter heterogeneity in Section 3. In Section 4, we explore how varying heterogeneity modifies bifurcations and varies the period of the collective oscillation. Sections 5 and 6 show how to deal with two and more, respectively, heterogeneous parameters. We conclude in Section 7.

## 2 The model

 Our illustrative example is a network of model neurons thought to describe at some level the dynamics of the pre-Bötzinger complex, governed by the following equations:

(1) $$C\frac{dV_{i}}{dt}=-g_{\mathrm{Na}}m(V_{i})h_{i}(V_{i}-V_{\mathrm{Na}})-g_{l}(V_{i}-V_{l})+I_{\mathrm{syn}}^{i}+I_{\mathrm{app}}^{i},$$

(2) $$\frac{dh_{i}}{dt}=\frac{h_{\infty }(V_{i})-h_{i}}{\tau (V_{i})}$$

for i=1,…,N, where

(3) $$I_{\mathrm{syn}}^{i}=\frac{g_{\mathrm{syn}}(V_{\mathrm{syn}}-V_{i})}{N}\sum_{j=1}^{N}s(V_{j}),$$

as considered in the work of Rubin and Terman [14]. Here, $V_{i}$ is the membrane potential of cell *i*, and $h_{i}$ is a channel state variable for neuron *i* that is governing the inactivation of persistent sodium. Equations 1 and 2 were derived from the model in the works of Butera *et al.*[13,24] by blocking currents responsible for action potentials. A similar model with N=2 was considered in the work of Rubin [28], and Dunmyre and Rubin [29] considered synchronisation in the case N=3, where one of the neurons was quiescent, another was tonically firing, and the third one could be either quiescent, tonically firing or bursting. The neurons are all-to-all coupled via the term $I_{\mathrm{syn}}^{i}$; when $g_{\mathrm{syn}}=0$ the neurons are uncoupled. The various functions involved in the model equations are the following:

(4) $$s(V)=\frac{1}{1+\exp[-(V+40)/5]},$$

(5) $$\tau (V)=\frac{1}{\epsilon \cosh[(V+44)/12]},$$

(6) $$h_{\infty }(V)=\frac{1}{1+\exp[(V+44)/6]},$$

(7) $$m(V)=\frac{1}{1+\exp[-(V+37)/6]}.$$

The functions τ(V)$h_{\infty }(V)$ and m(V) are a standard part of the Hodgkin-Huxley formalism [8], and synaptic communication is assumed to act instantaneously through the function s(V). The parameter values we use initially are $V_{\mathrm{Na}}=50$$g_{l}=2.4$$V_{l}=-65$$V_{\mathrm{syn}}=0$C=0.21ϵ=0.1$g_{\mathrm{syn}}=0.3$ and $g_{\mathrm{Na}}=2.8$.

 Note that the synaptic coupling is excitatory. These parameters are the same as that used in the work of Rubin and Terman [14] except that they [14] used ϵ=0.01 and $g_{l}=2.8$, and their function s(V) had a more rapid transition from approximately 0 to 1 as *V* was increased. These changes in parameter values were made to speed up the numerical integration of Equations 1 and 2, and the methods presented here do not depend on the particular values of these parameters.

**Fig. 1:**
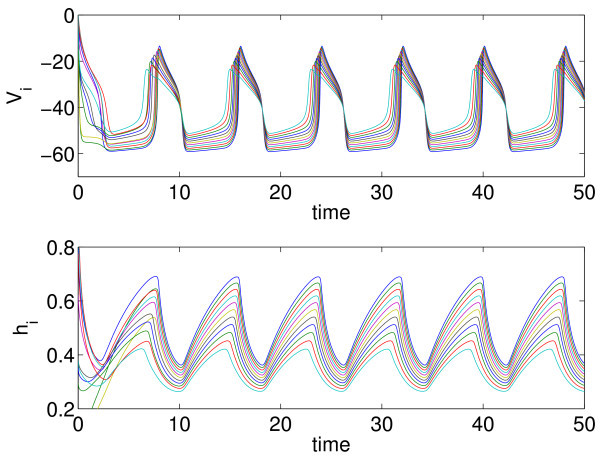
Solutions of Equations 1 and 2. These are the solutions when the $I_{\mathrm{app}}^{i}$ values are uniformly sampled from a uniform distribution on [10,25]. *Top*: $V_{i}$ as functions of time. *Bottom*: $h_{i}$ as functions of time N=101. Different line colours correspond to different neurons (only every 10th neuron is shown).

 If the values of the applied current $I_{\mathrm{app}}^{i}$ are taken from a uniform distribution on the interval [10,25], the behaviour is as shown in Figure 1. After a transient, we see a synchronous behaviour, *i.e.* all neurons oscillate periodically with the same period, although the heterogeneity in the $I_{\mathrm{app}}^{i}$ means that each neuron follows a slightly different periodic orbit in its own (V,h) phase space. (Because spiking currents have been removed in the derivation of Equations 1 and 2, these oscillations are interpreted as burst envelopes, *i.e.* neuron *i* is assumed to be spiking when $V_{i}$ is high and quiescent when $V_{i}$ is low.) It is this stable synchronous periodic behaviour that is of interest: In what parameter regions does it exist, and how does the period vary as parameters are varied? Butera *et al.*[13] observed that including parameter heterogeneity in a spiking model for the pre-Bötzinger complex, it increased both the range of parameters over which bursting occurred and the range of burst frequencies (this being functionally advantageous for respiration), and this was the motivation for the study of Rubin and Terman [14]. 

## 3 Managing heterogeneity

### 3.1 The continuum limit

**Fig. 2:**
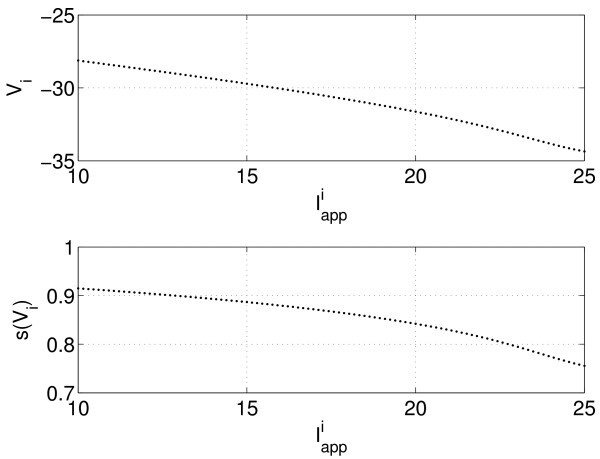
Solutions of Equations 1 and 2 at one instant in time. $V_{i}$ (*top*) and $s(V_{i})$ (*bottom*) as functions of $I_{\mathrm{app}}^{i}$, N=101. This shows a state where all neurons are active (see Figure 1). If the network was switching from active to quiescent or vice versa, there would be a steep ‘front’ where the $V_{i}$ changed rapidly with *i* although they would still form a continuous curve.

The key observation behind our approach can be seen in Figure 2, where we plot the $V_{i}$ and $s(V_{i})$ as functions of $I_{\mathrm{app}}^{i}$ at one instant in time. Once the neurons have synchronised, $V_{i}$ values (and $h_{i}$ and any smooth functions of these variables) appear to vary smoothly when plotted as a function of the heterogeneous parameter $I_{\mathrm{app}}^{i}$. This is also the case when the $I_{\mathrm{app}}^{i}$ values are chosen randomly from the interval [10,25] rather than uniformly (not shown). This suggests that in the limit of N→∞, at any instant in time, *V* and *h* will be smooth functions of the continuous variable $I_{\mathrm{app}}$. We now consider this case where $I_{\mathrm{app}}$ is a continuous random variable with a uniform density on the interval [10,25]. We parametrise $I_{\mathrm{app}}$ as $I_{\mathrm{app}}=I_{m}+I_{s}\mu$, where the probability density function for *μ* is as follows:

(8) $$p(\mu )=\{\begin{array}{cc} 1/2, & -1\leq \mu \leq 1,\\ 0, & \text{otherwise}. \end{array}$$

$V_{i}(t)$ and $h_{i}(t)$ become V(μ,t) and h(μ,t), respectively, and the points in Figure 2 ‘fill in’ to form continuous functions. In the given example, we had $I_{m}=17.5$ and $I_{s}=7.5$. Thus, the ordinary differential equations (ODEs) 1 and 2 become the following:

(9) $$\begin{array}{rcl} C\frac{\partial V(\mu ,t)}{\partial t} & = & -g_{\mathrm{Na}}m\left(V(\mu ,t)\right)h(\mu ,t)\left(V(\mu ,t)-V_{\mathrm{Na}}\right)-g_{l}\left(V(\mu ,t)-V_{l}\right)\\ & & +I_{\mathrm{syn}}(\mu ,t)+I_{m}+I_{s}\mu , \end{array}$$

(10) $$\frac{\partial h(\mu ,t)}{\partial t}=\frac{h_{\infty }(V(\mu ,t))-h(\mu ,t)}{\tau (V(\mu ,t))},$$

where

(11) $$I_{\mathrm{syn}}(\mu ,t)=g_{\mathrm{syn}}\left(V_{\mathrm{syn}}-V(\mu ,t)\right)\int_{-1}^{1}s\left(V(\mu ,t)\right)p(\mu )\;d\mu .$$

The results for N→∞ should provide a good approximation to the behaviour seen when *N* is large but finite, which is the realistic (although difficult to simulate) case. The continuum limit presented in this section was first introduced by Rubin and Terman [14], but their contribution was largely analytical, whereas ours will be largely numerical.

### 3.2 Stochastic Galerkin

 One approach to studying Equations 9 and 11, motivated by techniques developed in the context of uncertainty quantification [25,26], is to expand the functions V(μ,t) and h(μ,t) in orthogonal polynomials in *μ*, with the choice of particular polynomials determined by the probability density of *μi.e.* the distribution of the heterogeneous parameter. For the uniform density p(μ), one would choose Legendre polynomials, written as follows:

(12) $$V(\mu ,t)=\sum_{i=0}^{\infty }a_{i}(t)P_{i}(\mu ),\;h(\mu ,t)=\sum_{i=0}^{\infty }b_{i}(t)P_{i}(\mu ),$$

where $P_{i}$ is the *i*th Legendre polynomial; this is known as a ‘polynomial chaos’ expansion [3]. Substituting Equation 12 into Equation 9, multiplying both sides by $P_{j}(\mu )p(\mu )$ and integrating over *μ* between −1 and 1, the orthogonality properties of Legendre polynomials with uniform weight allows one to obtain the ODE satisfied by $a_{j}(t)$. Similarly, one can use Equation 10 to obtain the ODEs governing the dynamics of $b_{j}(t)$. Having solved (a truncated set of) these ODEs, one could reconstruct V(μ,t) and h(μ,t) using Equation 12. This is referred to as the stochastic Galerkin method [25]. However, the integrals just mentioned cannot be performed analytically. They must be calculated numerically at each time step in the integration of the ODEs for $a_{i}$ and $b_{i}$; this is computationally intensive. Note that the optimal choice of orthogonal polynomials is determined by the distribution of the heterogeneous parameter: for a uniform distribution, we use Legendre polynomials; for other distributions, other families of orthogonal polynomials are used [25,26].

### 3.3 Stochastic collocation

 An alternative, motivated by the stochastic collocation method [25], is to simply discretise in the *μ* direction, obtaining *N* different $\mu_{i}$ values, and then solve Equations 9 and 10 at each of the $\mu_{i}$, using the values of $s(V(\mu_{i},t))$ to approximate the integral in Equation 11.

It is important to realize that the number (*N*) of neurons simulated in this approach may well be much smaller than the number of neurons in the ‘true’ system, considered to be in the thousands. Notice also that these neurons are ‘mathematically’ coupled to one another via the discretisation of the integral (Equation 11), which is an approximation of the continuum limit.

Using the values of $s(V(\mu_{i},t))$ to approximate the integral in Equation 11, we are in fact including the influence of *all* other neurons (an infinite number of them in the continuum limit), not just those that we have retained in our reduced approximation. We now examine how different discretisation schemes affect several different calculations.

#### 3.3.1 Period calculation

**Fig. 3:**
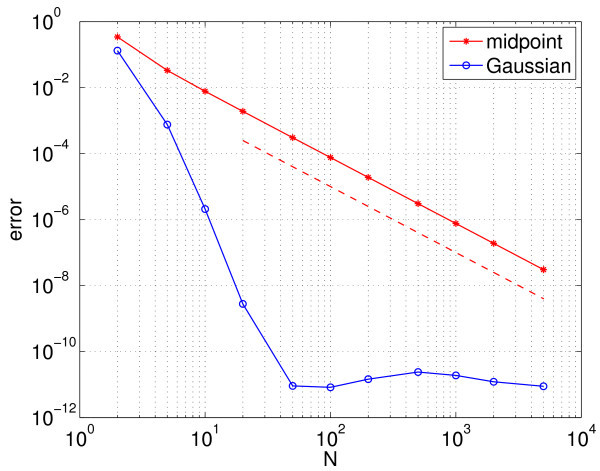
Error in the calculated period of the synchronised oscillators. Error in the calculated period of the synchronised oscillators as a function of the number of neurons simulated (*N*) for the midpoint rule (*red stars*) and the Gaussian quadrature (*blue circles*). Also shown (*dashed*) is a line corresponding to error approximately $N^{-2}$, to guide the eye.

 Firstly, we consider the period of the collective oscillations seen in Figure 1. The analogue of finite differences, or the method of lines, is to uniformly discretise the interval [−1,1] into *N* values, $\mu_{i}$, and to solve Equations 9 and 10 at each of the $\mu_{i}$. Defining $\mu_{i}=-1+2(i-1/2)/N$ for i=1,2,…,N, we approximate the integral in Equation 11 using the composite midpoint rule:

(13) $$\int_{-1}^{1}s\left(V(\mu ,t)\right)p(\mu )\;d\mu \approx \frac{1}{N}\sum_{i=1}^{N}s\left(V(\mu_{i},t)\right)$$

which, after defining $V_{i}(t)=V(\mu_{i},t)$, is nothing more than the sum in Equation 3, where $I_{\mathrm{app}}^{i}=I_{m}+I_{s}\mu_{i}$. To show convergence of the calculation of the period with *N*, we plot the error in Figure 3 with red stars; the error is defined to be the absolute value of the difference between the calculated period and the true period (defined below). We see that the error scales as $N^{-2}$ as expected from numerical analysis [30]. (All numerical integration was performed using Matlab’s ode113 with an absolute tolerance of 10^−10^ and a relative tolerance of 10^−12^.) 

 However, by choosing non-uniformly spaced values of $\mu_{i}$, we can evaluate the integral in Equation 13 much more accurately. (By ‘more accurately’, we mean either that for a fixed *N*, using the non-uniformly spaced $\mu_{i}$ will result in a smaller error than that obtained using uniform spacing, or that to obtain a fixed accuracy, using non-uniform spacing will require a smaller *N* than that needed for uniform spacing.) Specifically, for a fixed *N*, if we choose $\mu_{i}$ to be the *i*th root of $P_{N}(\mu )$, where $P_{N}$ is the *N*th Legendre polynomial, normalised so that $P_{N}(1)=1$, and the weights

(14) $$w_{i}=\frac{1}{(1-\mu_{i}^{2}){\left[P_{N}^{\prime }(\mu_{i})\right]}^{2}},$$

then the Gauss-Legendre quadrature rule [31] is

(15) $$\int_{-1}^{1}s\left(V(\mu ,t)\right)p(\mu )\;d\mu \approx \sum_{i=1}^{N}w_{i}s\left(V(\mu_{i},t)\right).$$

Convergence of the error in the period with *N* is shown in Figure 3 (blue circles), where we see the very rapid convergence expected from a spectral method. For 50≲N, the error in the period calculation using this method is dominated by errors in the numerical integration of the Equations 9 and 10 in time, rather than in the approximate evaluation of the integral in Equation 11. (The true period was calculated using the Gauss-Legendre quadrature with *N* significantly larger than 10^4^ and is approximately 8.040104851819.) The rapid convergence of the Gauss-Legendre quadrature is a consequence of the fact that the function s(V(μ)) is a sufficiently smooth function of *μ* (see Figure 2). This smoothness will arise only when the oscillators become fully synchronised.

#### 3.3.2 Hopf bifurcations

**Fig. 4:**
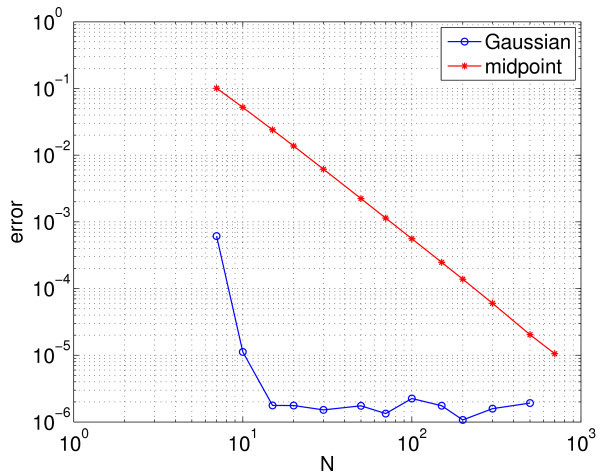
Error in calculation error of the value of $I_{m}$ where upper Hopf bifurcation occurs. Error in the calculation of the value of $I_{m}$ at which the upper Hopf bifurcation occurs using the midpoint rule (*red stars*) and the Gaussian quadrature (*blue circles*). Other parameters: $g_{\mathrm{syn}}=0.3$, $I_{s}=7.5$. The midpoint rule error decays as $1/N^{2}$. For 10<N, the error using the Gaussian quadrature is dominated by the precision with which the Hopf bifurcation can be located, hence the plateau.

By decreasing or increasing $I_{m}$ (the mean of the $I_{\mathrm{app}}^{i}$), we find that the oscillations in Figure 1 terminate in Hopf bifurcations. We now examine the effects of the different discretisations mentioned on the detection of these Hopf bifurcations. In Figure 4, we see the error in calculating the value of $I_{m}$ at which the upper Hopf bifurcation occurs as a function of *N*, the number of points used, for the two different schemes (the true value, again calculated using the Gauss-Legendre quadrature with a large *N*, is approximately $I_{m}=33.1262$). 

**Fig. 5:**
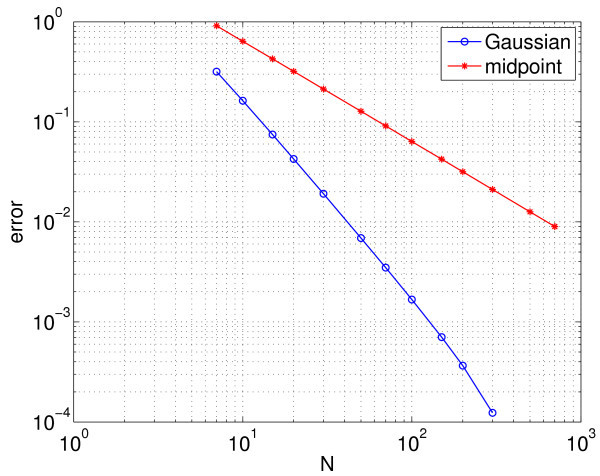
Error in calculation error of the value of $I_{m}$ where lower Hopf bifurcation occurs. Error in calculation of the value of $I_{m}$ at which the lower Hopf bifurcation occurs using the midpoint rule (*red stars*) and the Gaussian quadrature (*blue circles*). The error for the midpoint rule appears to decay as 1/N. Other parameters: $g_{\mathrm{syn}}=0.3$, $I_{s}=7.5$.

The expected behaviour (very rapid convergence for Gaussian quadrature and the error scaling as $N^{-2}$ for the composite midpoint rule) is seen (as compared with Figure 3). Figure 5 shows a similar calculation but for the lower Hopf bifurcation which occurs at $I_{m}\approx 6.064$. Several interesting points in contrast with the results in Figure 4 are evident: The error in the composite midpoint rule appears to decay as $N^{-1}$, while the error using the Gaussian quadrature appears to decay as $N^{-2}$. The reason for these differences is not clear. 

### 3.4 Summary

In this section, we have shown that a judicious choice of the values of the heterogeneous parameter, combined with a scheme for the Gaussian quadrature, allows us to calculate quantities of interest (such as the period of oscillation and the parameter value at which a Hopf bifurcation occurs) much more parsimoniously than a naive implementation of uniformly spaced $I_{i}$ values for a uniform distribution. Effectively, we have simulated the behaviour of a large network of oscillators by actually simulating a much smaller one, carefully choosing *which* oscillators to simulate (and how to couple them so as to also capture the effect of the omitted ones).

Having demonstrated this, we now fix N=10 and use the quadrature rule given in Equation 15. Note that our discretisation in *μ* can be thought of in two different ways. Firstly, we can consider the continuum limit (N→∞) as the true system, whose dynamics will be close to the real system which consists of a large number of neurons. Our scheme is then an efficient way of simulating this true system. The other interpretation is that the true system consists of a large, finite number of neurons with randomly distributed parameter(s), and our scheme is a method for simulating such a system but using far fewer oscillators.

In the next section, we investigate the effects of varying $I_{m}$, $I_{s}$ and $g_{\mathrm{syn}}$. In a later section, we consider more than one heterogeneous parameter and show how tensor product grids and sparse tensor product grids can be used to accurately calculate the effects of further, independently distributed, heterogeneities.

## 4 The effects of heterogeneity

### 4.1 A single neuron

**Fig. 6:**
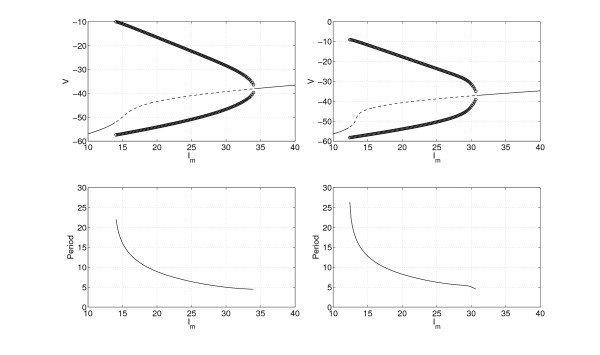
The bifurcation behaviour, *V* as functions of $I_{m}$ and period of the stable periodic orbit. *Left*: the bifurcation behaviour of a single uncoupled neuron (N=1, $g_{\mathrm{syn}}=0$). *Top left*: voltage *V* at a fixed point (*solid*, stable; *dashed*, unstable) and the maximum and minimum of *V* over one period of oscillation (*circles*), as a function of $I_{m}$. *Bottom left*: period of the stable periodic orbit for a single uncoupled neuron. The apparent discontinuity in the periodic orbit towards low $I_{m}$ is because of the canard nature of the oscillations (mentioned in the text). *Right*: the bifurcation behaviour of a single self-coupled neuron (N=1, $g_{\mathrm{syn}}=0.3$). *Top right*: voltage *V* at a fixed point (*solid* stable, *dashed* unstable) and the maximum and minimum of *V* over one period of oscillation (*circles*), as a function of $I_{m}$. *Bottom right*: period of the stable periodic orbit for a single self-coupled neuron.

 In order to investigate the effects of heterogeneity, we first examine a single uncoupled neuron (*i.e.*N=1 and $g_{\mathrm{syn}}=0$). The behaviour as $I_{m}$ is varied as shown in Figure 6 (left panel). For this range of $I_{m}$, there is always one fixed point, but it undergoes two Hopf bifurcations as $I_{m}$ is varied, leading to a family of stable periodic orbits. The period decreases monotonically with increasing $I_{m}$. The lower Hopf bifurcation results in a canard periodic solution [32] which very rapidly increases in amplitude as $I_{m}$ is increased. This is related to the separation of time scales between the *V* dynamics (fast) and the *h* dynamics (slow). In the left panel of Figure 6, we see that some of the neurons in the network whose behaviour is shown in Figure 1 would be quiescent *when uncoupled*, while most would be periodically oscillating. 

**Fig. 7:**
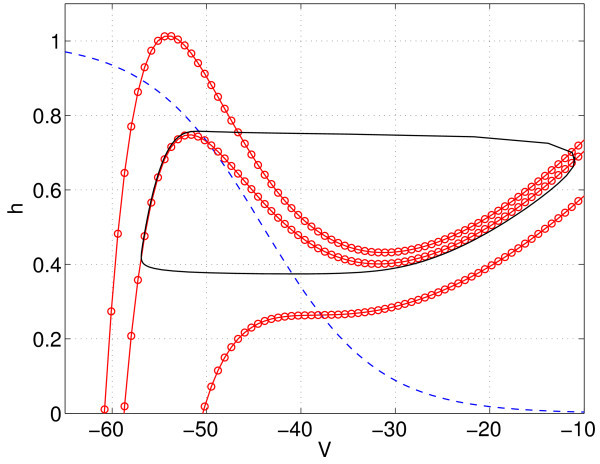
The phase plane for a single uncoupled neuron. *h*-nullcline (*dashed*, on which dh/dt=0) and the *V*-nullclines (*circles*, on which dV/dt=0) for $I_{m}=10,15\text{ and }35$ (*top* to *bottom*). Also shown (*solid*) is the stable periodic orbit that exists when $I_{m}=15$.

The behaviour in the left panel of Figure 6 can also be understood by looking at the (V,h) phase plane for different values of $I_{m}$ - see Figure 7. The behaviour of one self-coupled neuron (N=1, $g_{\mathrm{syn}}=0.3$) is shown in Figure 6 (right panel). We see that the main effect of self-coupling is to move both Hopf bifurcations to lower values of $I_{m}$. 

### 4.2 A coupled population of neurons

**Fig. 8:**
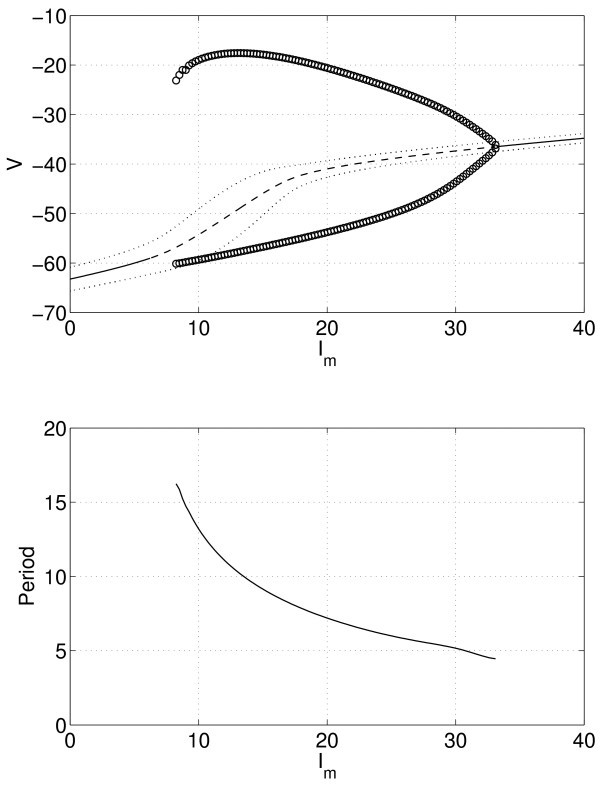
The bifurcation behaviour of a heterogeneous population. *Top*: mean voltage at a fixed point (*solid* stable, *dashed* unstable), mean ± one standard deviation (*dotted*), and the maximum and minimum of the mean of *V* over one period of oscillation (*circles*), as a function of $I_{m}$. *Bottom*: period of the stable periodic orbit. N=10, $g_{\mathrm{syn}}=0.3$, $I_{s}=7.5$.

Now, consider a coupled heterogeneous population with N=10 neurons. Parameter values are $g_{\mathrm{syn}}=0.3$ and $I_{s}=7.5$. (Note that if $I_{s}=0$, we recover the results for one self-coupled neuron.) The results from varying $I_{m}$ are shown in Figure 8. Comparing with the right panel of Figure 6, we see that including heterogeneity widens the range of $I_{m}$ values for which oscillations occur. The periodic orbit cannot be followed below $I_{m}\approx 8$, as more complex oscillations than purely periodic occur (not shown), as discussed below. Note that the mean voltage at the fixed point is easily calculated as $\bar{V}\equiv \sum_{i=1}^{10}w_{i}V_{i}$, where $V_{i}$ is the steady state value of neuron *i*, and the variance of the $V_{i}$’s is simply $\sum_{i=1}^{10}w_{i}{\left(V_{i}-\bar{V}\right)}^{2}$. (Recall that the weights $w_{i}$ are given in Equation 14.) 

**Fig. 9:**
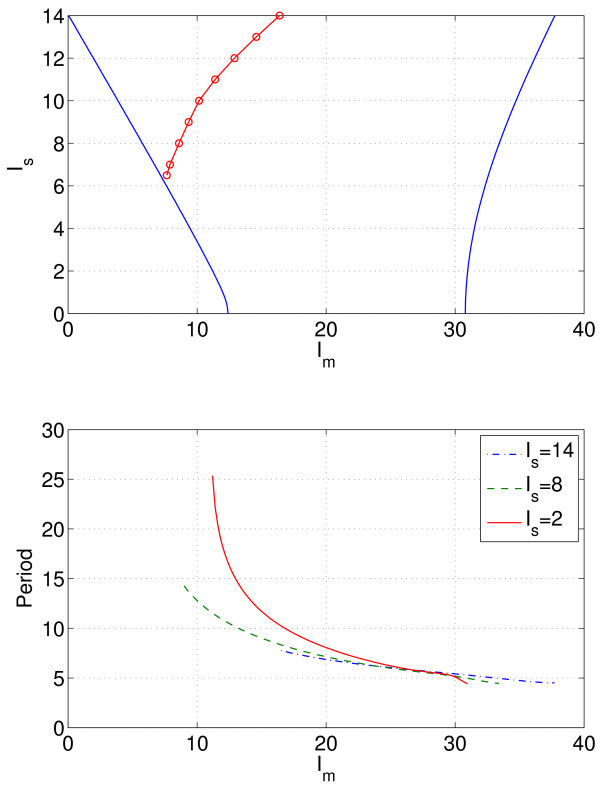
Hopf bifurcation curves and period of the stable periodic orbit for three different values of $I_{s}$. *Top*: Hopf bifurcation curves (*solid*) and the curve on which the periodic orbit created in the rightmost Hopf bifurcation loses stability (*circles*, found from direct simulation). *Bottom*: period of the stable periodic orbit for three different values of $I_{s}$, the spread of the heterogeneity. For $I_{s}=8\text{ and }14$, the curves are terminated at low $I_{m}$ when the periodic orbit loses stability to a more complex oscillation. $g_{\mathrm{syn}}=0.3$, N=10.

To better understand these results, we can follow the Hopf bifurcations as *two* parameters are varied. Figure 9 (top) shows the two curves of Hopf bifurcations in the $I_{m}$, $I_{s}$ plane for $g_{\mathrm{syn}}=0.3$. Increasing the ‘spread’ of the heterogeneity, *i.e.* increasing $I_{s}$, increases the range of values of $I_{m}$ for which periodic oscillations are possible (between the Hopf bifurcations), but there may not necessarily exist stable periodic orbits over the entire range. For $I_{s}$ larger than about 6, *i.e.* for very heterogeneous neurons, the synchronous behaviour created in the rightmost Hopf bifurcation shown in Figure 9 (top) breaks up as $I_{m}$ is decreased at constant $I_{s}$, leading to complex oscillations (not shown). The break-up of the synchronous behaviour always involves the neurons with the lowest values of *μ*, *i.e.* the lowest values of $I_{\mathrm{app}}$. The curve in Figure 9 (top) where synchronous behaviour breaks up was found by slowly decreasing $I_{m}$ at constant $I_{s}$ until the break-up was observed. In principle, it could be found by numerical continuation of the stable periodic orbit created in the rightmost Hopf bifurcation, monitoring the orbit’s stability. 

**Fig. 10:**
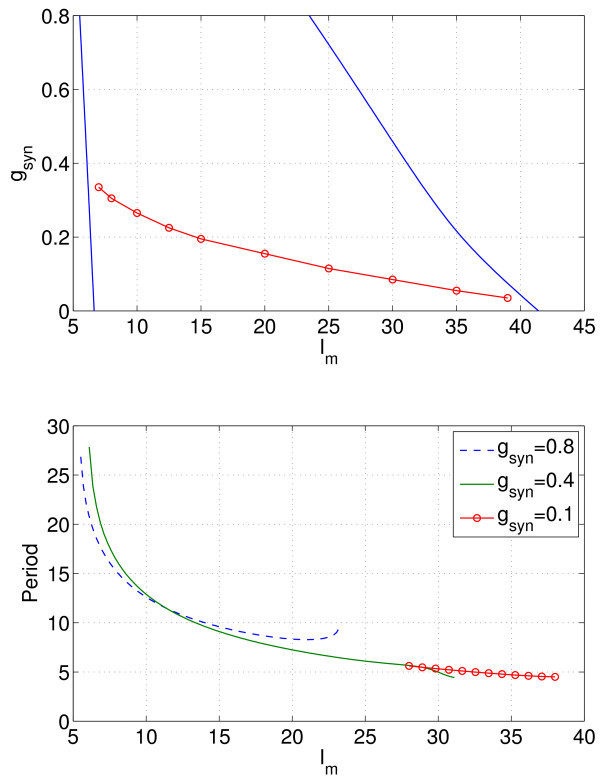
Hopf bifurcation curves and period of the stable periodic orbit for three different values of $g_{\mathrm{syn}}$. *Top*: Hopf bifurcation curves (*solid*) and the curve on which the periodic orbit created in the rightmost Hopf bifurcation loses stability (*circles*, obtained by direct simulation). Synchronous oscillations occur only *above* the curve shown with *red circles*. *Bottom*: period of the stable periodic orbit for three different values of $g_{\mathrm{syn}}$. The curve for $g_{\mathrm{syn}}=0.1$ is terminated at low $I_{m}$ when the periodic orbit loses stability to a more complex oscillation. $I_{s}=7.5$, N=10.

Now, consider varying $g_{\mathrm{syn}}$ and $I_{m}$ for a fixed $I_{s}=7.5$. As seen in Figure 10, the range of values of $I_{m}$ for which oscillations may arise decreases at $g_{\mathrm{syn}}$ increases (both Hopf bifurcations move to lower values of $I_{m}$), and for small $g_{\mathrm{syn}}$ (*i.e.* weak coupling), the neurons are no longer synchronous, due to break-up as discussed. The conclusion is that, in order to obtain robust synchronous oscillations, we need moderate to large coupling ($g_{\mathrm{syn}}$) and a not-too-heterogeneous population ($I_{s}$ not too large). This is perhaps not surprising, but our main point here is to demonstrate how the computation of the effects of heterogeneity can easily be accelerated. We now consider more than one heterogeneous parameter. 

## 5 Two heterogeneous parameters

Now, consider the case where both $I_{\mathrm{app}}$ and $g_{\mathrm{Na}}$ for each neuron are randomly (independently) distributed. We keep the uniform distribution for the $I_{\mathrm{app}}$, choosing $I_{m}=25$, $I_{s}=7.5$ so that the $I_{\mathrm{app}}$ come from a uniform distribution on [17.5,32.5]. We choose the $g_{\mathrm{Na}}$ from a normal distribution with a mean of 2.8, and standard deviation *σ* and set $g_{\mathrm{syn}}=0.3$. We keep 10 points in the *μ* direction and use the values of $\mu_{i}$ and $w_{i}$ from above to perform integration in the *μ* direction. The quantity *M* refers to the number of different $g_{\mathrm{Na}}$ values chosen, and we thus simulate 10*M* appropriately as coupled neurons.

The values of $I_{\mathrm{app}}$ and $g_{\mathrm{Na}}$ for the different neurons are selected based on the tensor product of the vectors formed from $I_{\mathrm{app}}$ and $g_{\mathrm{Na}}$. Similarly, the weights in a sum of the form (Equation 15) will be formed from a tensor product of the $w_{i}$ associated with the $I_{\mathrm{app}}$ direction and those associated with the $g_{\mathrm{Na}}$.

We initially choose σ=0.25 and write $g_{\mathrm{Na}}=2.8+\sigma \lambda$, where *λ* has the probability density function

(16) $$q(\lambda )=\frac{1}{\sqrt{2\pi }}e^{-\lambda^{2}/2},$$

*i.e.λ* is normally distributed. Then, as mentioned, the continuum variables *V* and *h* are written in the form V(μ,λ,t) and h(μ,λ,t), respectively, and the sum in Equation 3 becomes

(17) $$\int_{-\infty }^{\infty }\int_{-1}^{1}s\left(V(\mu ,\lambda ,t)\right)p(\mu )q(\lambda )\;d\mu \;d\lambda .$$

Keeping the Gauss-Legendre rule in the *μ* direction, this gives

(18) $$\int_{-\infty }^{\infty }\sum_{i=1}^{10}w_{i}s\left(V(\mu_{i},\lambda ,t)\right)q(\lambda )\;d\lambda .$$

The simplest approach to this integral is the Monte Carlo method [30], where we simply randomly choose *M* values of *λ* from the unit normal distribution and calculate an approximation to the integral as the following:

(19) $$\frac{1}{M}\sum_{j=1}^{M}\sum_{i=1}^{10}w_{i}s\left(V(\mu_{i},\lambda_{j},t)\right).$$

**Fig. 11:**
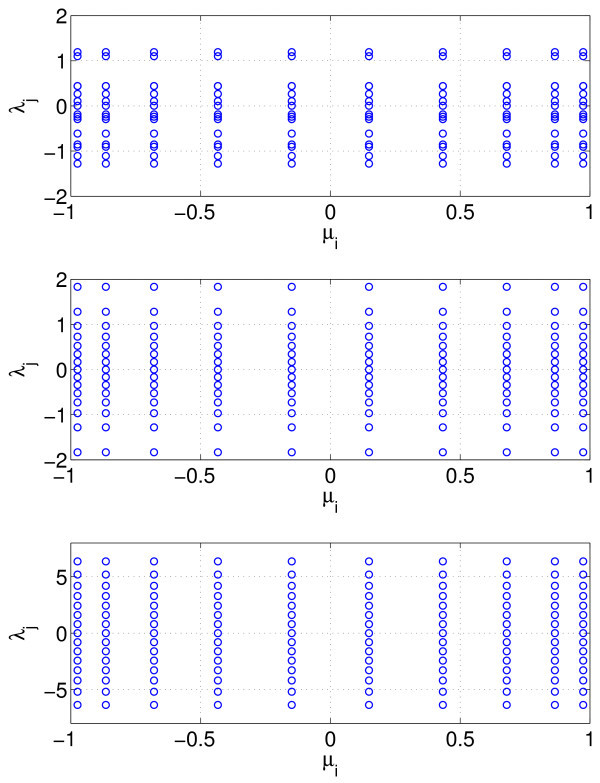
Examples of the heterogeneity grid values of $\mu_{i}$ and $\lambda_{j}$ for M=15. *Top*: the $\lambda_{j}$ values are randomly chosen from a unit normal distribution. *Middle*: the $\lambda_{j}$ values are chosen by uniformly sampling the inverse cumulative distribution function of a unit normal distribution. *Bottom*: the $\lambda_{j}$ values are the roots of $H_{15}$, the 15th Hermite polynomial. In all cases, the $\mu_{i}$ are roots of $P_{10}$, the 10th Legendre polynomial.

Here, the weights in the *λ* direction are all equal to 1/M. An example of the $\mu_{i}$ and $\lambda_{j}$ for M=15 is shown in Figure 11 (top). Another approach is to transform the integral to one over [0,1] and use the composite midpoint rule on that new variable. Specifically, if we define

(20) $$z=Q(\lambda )\equiv \int_{-\infty }^{\lambda }q(s)\;ds,$$

*i.e.Q* is the cumulative density function for *λ*, and then for a general function *f*, the integral

(21) $$\int_{-\infty }^{\infty }f(\lambda )q(\lambda )\;d\lambda$$

can be written as

(22) $$\int_{0}^{1}f\left(Q^{-1}(z)\right)\;dz.$$

Thus, we define

(23) $$\lambda_{j}=Q^{-1}\left(\frac{j}{M}-\frac{1}{2M}\right),$$

for j=1,…,M and use the approximation (Equation 19). An example of the $\mu_{i}$ and $\lambda_{j}$ for M=15 is shown in Figure 11 (middle). It is better still to use the Gaussian quadrature (specifically, the Gauss-Hermite quadrature) in the *λ* direction. We approximate the integral

(24) $$\int_{-\infty }^{\infty }f(\lambda )q(\lambda )\;d\lambda \approx \sum_{j=1}^{N}v_{j}f(\lambda_{j}),$$

where $\lambda_{j}$ is the *j*th root of $H_{N}$; the *N*th ‘probabilists’ Hermite polynomial’ and the weights $v_{j}$ are given by

(25) $$v_{j}=\frac{N!}{{\left[NH_{N-1}(\lambda_{j})\right]}^{2}}.$$

(The first few probabilists’ - as opposed to physicists’ - Hermite polynomials are $H_{0}(x)=1$, $H_{1}(x)=x$, $H_{2}(x)=x^{2}-1,\ldots$ .) Thus, we approximate the integral in Equation 17 by the double sum:

(26) $$\int_{-\infty }^{\infty }\int_{-1}^{1}s\left(V(\mu ,\lambda ,t)\right)p(\mu )q(\lambda )\;d\mu \;d\lambda \approx \sum_{j=1}^{M}\sum_{i=1}^{10}v_{j}w_{i}s\left(V(\mu_{i},\lambda_{j},t)\right).$$

An example of the $\mu_{i}$ and $\lambda_{j}$ for M=15 is shown in Figure 11 (bottom).

**Fig. 12:**
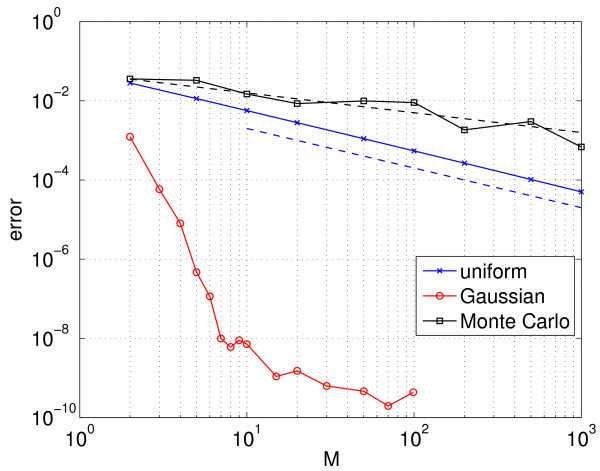
Error in the calculated period using three different methods (see text) for σ=0.25. The *dashed lines*, drawn to guide the eye, have slopes −1/2 (*upper*) and −1 (*lower*). For the Monte Carlo simulations, the average of 10 calculations for each *M* is shown.

The result of using these three different methods to allocate the $g_{\mathrm{Na}}$ (and thus, to select the reduced number of appropriately coupled neurons we simulate) is shown in Figure 12. This figure shows the error in the calculated period as *M* is varied. (The true period was calculated using the Gauss-Hermite quadrature with a large *M* in the $g_{\mathrm{Na}}$ direction.) 

We see that as expected, the Gauss-Hermite quadrature performs the best, with the error saturating between M=10 and M=20. (Recalling that we are using 10 points in the *μ* direction, this is consistent with the idea that roughly the same number of points should be used in each random direction.) Using the Monte Carlo method, *i.e.* randomly choosing, the $g_{\mathrm{Na}}$ gives convergence that scales as $M^{-1/2}$. Uniformly sampling the inverse cumulative distribution function gives an error that appears to scale as $M^{-1}$. This is at variance with the expected scaling of $M^{-2}$ for the composite midpoint rule applied to a function with a bounded second derivative, but the inverse CDF of a normal distribution (*i.e.*$Q^{-1}(z)$) does not have a bounded second derivative, and an error analysis of Equation 22 (not shown) predicts a scaling of $M^{-1}$, as observed.

## 6 Sparse grids

The process described above can obviously be generalised to more than two randomly, but independently, distributed parameters. The distribution of each parameter determines the type of quadrature which should be used in that direction, and the parameter values and weights are formed from tensor products of the underlying one-dimensional rules. However, the curse of dimensionality will restrict how many random parameters can be accurately sampled. If we use *N* points in each of *D* random dimensions, the number of neurons we need to simulate is $N^{D}$.

**Fig. 13:**
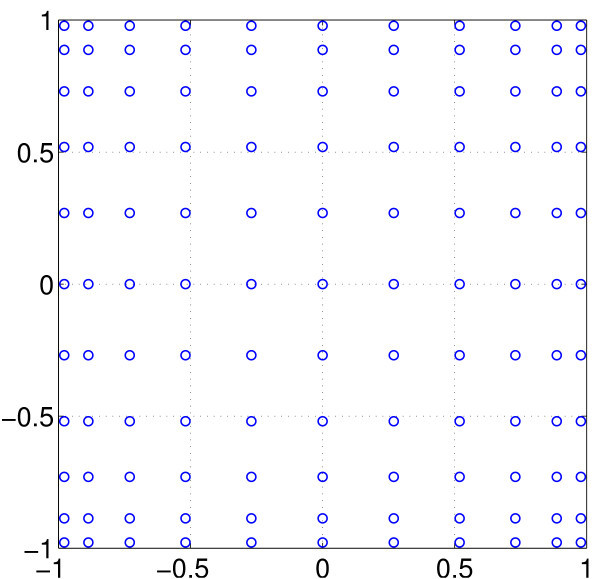
Full tensor product using 11 points in each direction (121 points in total). The points are the roots of $P_{11}$, the 11th Legendre polynomial.

 One way around this problem is to use sparse grids [33,34], as introduced by Smolyak [35]. The basic idea is to use sparse tensor products, chosen in such a way as to have similar accuracy to the corresponding full tensor product, but with fewer grid points, and thus (in our case) fewer neurons to simulate. A general theory exists [33,34], but to illustrate the idea, suppose we have two uncorrelated random parameters, each is distributed uniformly between −1 and 1. A full tensor product for the Gauss-Legendre quadrature using 11 points in each direction is shown in Figure 13. 

To form a two-dimensional sparse grid using the Gauss-Legendre quadrature, we first write the one-dimensional integration rule for integrating a function *f* as

(27) $$\int_{-1}^{1}f(x)\;dx\approx U^{i}(f)\equiv \sum_{j=1}^{N_{i}}w_{j}f(x_{j}),$$

where i∈N; $w_{j}$ are the weights, and $x_{j}$ are the nodes. We form a nested family of such rules with index *i* where the correspondence between *i* and $N_{i}$ is given in the following: 

*i.e.*$N_{i}=2^{i+1}-1$. Then, the level *L* rule in two spatial dimensions is

(28) $$A(L,2)=\sum_{|i|=L}\left(U^{i_{1}}⊗U^{i_{2}}\right)-\sum_{|i|=L-1}\left(U^{i_{1}}⊗U^{i_{2}}\right),$$

where $i\in N^{2}$ and $|i|=i_{1}+i_{2}$. The approximation of the integral of *f* over the domain ${\left[-1,1\right]}^{2}$ is A(L,2)(f). So for example, the level 2 rule (in 2 spatial dimensions and using Gauss-Legendre quadrature) is

(29) $$A(2,2)=U^{0}⊗U^{2}+U^{1}⊗U^{1}+U^{2}⊗U^{0}-\left(U^{0}⊗U^{1}+U^{1}⊗U^{0}\right).$$

**Fig. 14:**
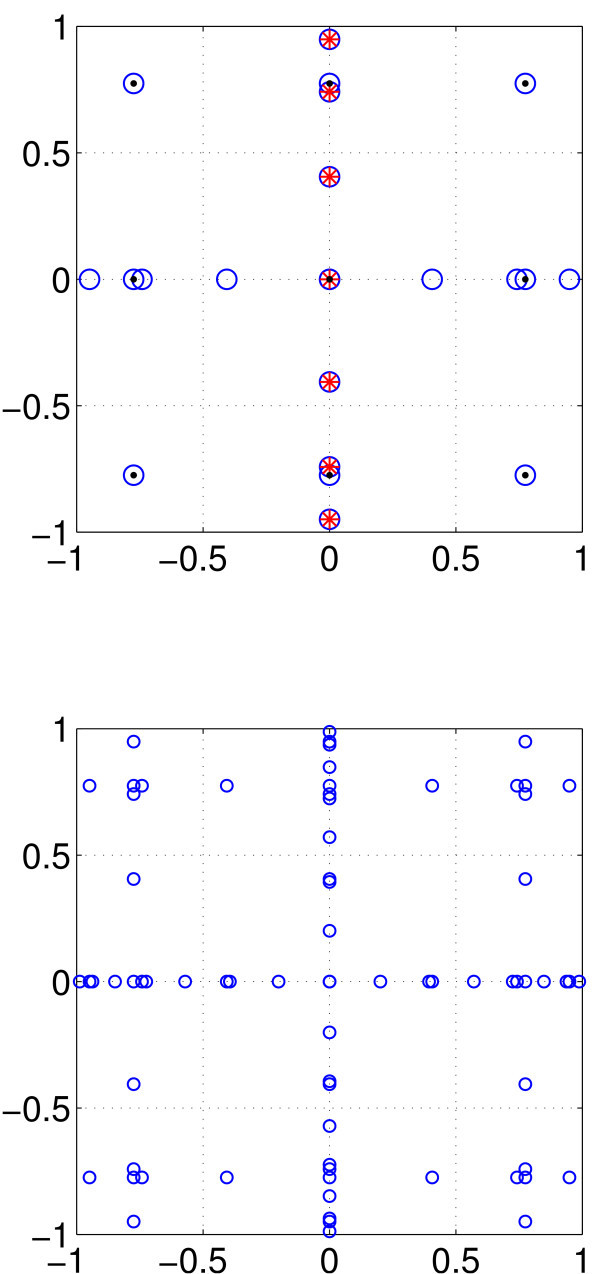
Grids for rules A(2,2) and A(3,2). *Top*: *blue circles*: the grid for rule A(2,2) (*i.e.* level 2 in 2 spatial dimensions) using Gauss-Legendre quadrature. *Red crosses*: grid corresponding to $U^{0}⊗U^{2}$ (one point horizontally, 7 vertically). *Black dots*: grid corresponding to $U^{1}⊗U^{1}$ (3 points both horizontally and vertically). The three black dots on the *y*-axis correspond to $U^{0}⊗U^{1}$, while the three black dots on the *x*-axis correspond to $U^{1}⊗U^{0}$. *Bottom*: the grid for rule A(3,2) (*i.e.* level 3 in 2 spatial dimensions). Rule A(2,2) has 21 grid points, and rule A(3,2) has 73.

The grid for this rule is shown in Figure 14 (top), along with grids corresponding to several of its components.^a^ Figure 14 (bottom) shows the grid for rule A(3,2). 

**Fig. 15:**
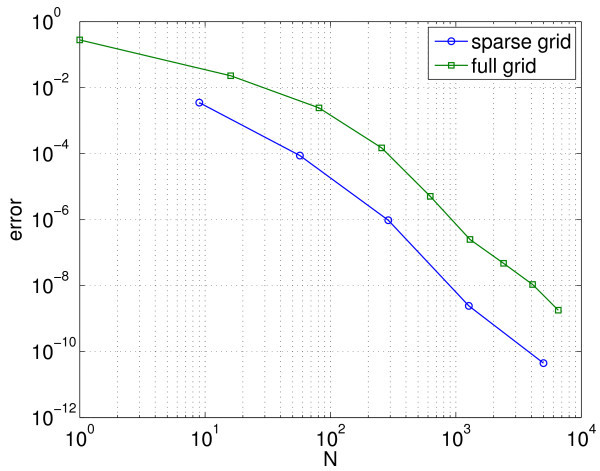
Error in calculation of period. This happens when four distinct parameters are simultaneously heterogeneous (independently of one another) for both full and sparse grids. See text for details. *N* is the number of neurons simulated.

Rules such as these can be constructed for an arbitrary number of spatial dimensions, using a variety of quadrature rules (and possibly different rules in different dimensions). Their advantage becomes apparent as the dimension of the space to be integrated over (or in our case, the number of heterogeneous parameters) is increased. To illustrate this, we consider as an example the model, Equations 1 and 2 with $I_{\mathrm{app}}$ uniformly spread between 17.5 and 32.5; the $g_{\mathrm{Na}}$ uniformly spread between 2.55 and 3.05; $V_{\mathrm{syn}}$ uniformly spread between −1 and 1; and $V_{\mathrm{Na}}$ uniformly spread between 49 and 51, *i.e.* 4 independent random dimensions. A comparison of the error in calculating the period of collective oscillation using full and sparse grids is shown in Figure 15. 

We see that for fixed *N*, the sparse grid calculation is approximately two orders or magnitude more accurate than the full grid - implying, in turn, that *the way* we select the reduced number of neurons we retain to simulate the full system is critical. This relative advantage is expected to increase as the number of distributed parameters increases. As an example of the growth in the number of grid points, a level 6 calculation in 10 dimensions uses fewer than one million points, and the resulting system can be easily simulated on a desktop PC. (Note that the grid points and weights are calculated before the numerical integration starts, so the computational cost in producing data like that shown in Figure 15 is almost entirely due to numerical integration of the ODEs, which is proportional to the number of grid points, *i.e.* neurons, used.)

## 7 Discussion

In this paper, we have presented and demonstrated the use of a computationally efficient method for systematically investigating the effects of heterogeneity in the parameters of a coupled network of neural oscillators. The method constitutes a model reduction approach: By only considering oscillators with parameter values given by roots of families of orthogonal polynomials (Legendre, Hermite, …), we can use the Gaussian quadrature to accurately evaluate the term coupling the oscillators, which can be thought of as the discretisation of an integral over the heterogeneous dimension(s).

Effectively, we are simulating the behaviour of an infinite number of oscillators by only simulating a small number of judiciously selected ones, modifying appropriately the way they are coupled. When the oscillators are synchronised, or at a fixed point, the function to be integrated is a smooth function of the heterogeneous parameter(s), and thus, convergence is very rapid. The technique is general (although subject to the restriction immediately above) and can be used when there is more than one heterogeneous parameter, via full or sparse tensor products in parameter space. For a given level of accuracy, we are simulating far fewer neurons than might naively be expected. The emphasis here has been on computational efficiency rather than a detailed investigation of parameter dependence.

 The model we considered involved coupling only through the mean of a function, *s*, of the variable $V_{i}$ which, in the limit N→∞, can be thought of as an integral or, more generally, as a functional of V(μ). Thus, the techniques demonstrated here could also be applied to networks coupled through terms which, in the continuum limit, are integrals or functions of integrals. A simple example is diffusive coupling [3]; another possibility is coupling which is dependent upon the correlation between some or all of the variables. As mentioned, the technique will break down once the oscillators become desynchronised, as the dependence of state on parameter(s) will no longer be smooth. However, if the oscillators form several clusters [14,36], it may be possible to apply the ideas presented here to each cluster, as the dependence of state on parameter(s) *within* each cluster should still be smooth. Ideally, this reparametrisation would be done adaptively as clusters form, in the same way that algorithms for numerical integration adapt as the solution varies [30]. Alternatively, if a single oscillator ‘breaks away’ [27], the methods presented here could be used on the remaining synchronous oscillators, with the variables describing the state of the rogue oscillator also fully resolved. More generally, there are systems in which it is not necessarily the *state* of an oscillator that is a smooth function of the heterogeneous parameter, but the *parameters describing the distribution of states*[37,38], and the ideas presented here could also be useful in this case.

 The primary study with which we should compare our results is that of Rubin and Terman [14]. They considered essentially the same model as Equations 1 and 2 but with heterogeneity only in the $I_{\mathrm{app}}$ and, taking the continuum limit, referred to the curve in (V,h) space describing the state of the neurons at any instant in time as a ‘snake’. By making various assumptions, such as an infinite separation of time scales between the dynamics of the $V_{i}$ and the $h_{i}$, and that the dynamics of the $h_{i}$ in both the active and quiescent phases is linear, they derived an expression for the snake at one point in its periodic orbit and showed that such a snake is unique and stable. They also estimated the parameter values at which the snake ‘breaks’ and some oscillators lose synchrony. In contrast with their mainly analytical study, ours is mostly numerical and thus does not rely on any of the assumptions just mentioned. Using the techniques presented here, we were able to go beyond the work of Rubin and Terman, exploring parameter space.

 Our approach can be thought of as a particular parametrisation of this snake, which takes into account the probability density of the heterogeneity parameter(s); we also showed a systematic way of extending this one-dimensional snake to two and higher dimensions. Another paper which uses some of the same ideas as presented here is that of Laing and Kevrekidis [3]. There, the authors considered a finite network of coupled oscillators and used a polynomial chaos expansion of the same form as Equation 12. However, instead of integrating the equations for the polynomial chaos coefficients directly, they used projective integration [39] to do so, in an ‘equation-free’ approach [40] in which the equations satisfied by the polynomial chaos coefficients are never actually derived. They also chose the heterogeneous parameter values randomly from a prescribed distribution and averaged over realisations of this process in order to obtain ‘typical’ results. Similar ideas had been explored earlier by Moon *et al.*[27], who considered a heterogeneous network of phase oscillators.

 Assisi *et al.*[22] considered a heterogeneous network of coupled neural oscillators, deriving equations of similar functional form to Equations 9 and 11. Their approach was to expand the variables in a way similar to Equation 12 but using a small number of arbitrarily chosen ‘modes’ rather than orthogonal polynomials. Their choice of modes, along with the fact that their neural model consisted of ODEs with polynomial right hand sides, allowed them to analytically derive the ODEs satisfied by the coefficients of the modes. This approach allowed them to qualitatively reproduce some of the behaviour of the network such as the formation of two clusters of oscillators. However, in the general case modes should be chosen as orthogonal polynomials, the specific forms of which are determined by the distribution of the heterogeneous parameter(s) [25,26].

 The network we considered was all-to-all coupled, and the techniques presented should be applicable to other similar systems. The only requirement is that the relationship between the heterogeneity parameter(s) and the state of the system (possibly after transients) be smooth (or possibly piecewise smooth). An interesting extension is the case when the network under consideration is not all-to-all. Then, the effects of degree distribution may affect the dynamics of individual oscillators [38,41,42], and if we have a way of parameterising this type of heterogeneity, it might be possible to apply the ideas presented here to such networks. Degree distribution is a discrete variable, and corresponding families of orthogonal polynomials exist for a variety of discrete random variables [25,26].

## Competing interests

The authors declare that they have no competing interests.

## Authors’ contributions

CRL, YZ and BS performed calculations relating to the results presented. CRL and IGK wrote the manuscript. All authors read and approved the final manuscript.
